# Conservative therapy for the treatment of patients with somatic tinnitus attributed to temporomandibular dysfunction: study protocol of a randomised controlled trial

**DOI:** 10.1186/s13063-018-2903-1

**Published:** 2018-10-12

**Authors:** Sarah Michiels, Annemarie Christien van der Wal, Evelien Nieste, Paul Van de Heyning, Marc Braem, Corine Visscher, Vedat Topsakal, Annick Gilles, Laure Jacquemin, Marianne Hesters, Willem De Hertogh

**Affiliations:** 1 Department of Rehabilitation Sciences and Physiotherapy, Faculty of Medicine and Health Sciences, University of Antwerp, Antwerp, Belgium; 2 Department of Otorhinolaryngology, Antwerp University Hospital, Wilrijkstraat 10, 2650 Edegem, Belgium; 3 Faculty of Medicine and Health Sciences, University of Antwerp, Antwerp, Belgium; 4 Multidisciplinary Motor Centre Antwerp, University of Antwerp, Antwerp, Belgium; 5 Department of Translational Neurosciences, Faculty of Medicine and Health Sciences, University of Antwerp, Antwerp, Belgium; 6 Lab Dental Materials, University of Antwerp, 2610 Antwerp, Belgium; 7 Department of Special Care Dentistry, University Hospital Antwerp, 2650 Edegem, Belgium; 8 Faculty of Medicine and Health Sciences, University of Antwerp, 2610 Antwerp, Belgium; 9 Department of Oral Health Sciences, Academic Centre for Dentistry Amsterdam, University of Amsterdam and VU University Amsterdam, Research Institute MOVE Amsterdam, Amsterdam, Netherlands; 10 Department of Human and Social Welfare, University College Ghent, Ghent, Belgium

**Keywords:** Occlusal splints, Temporomandibular disorders, Physical therapy modalities, Somatic tinnitus, Somatosensory

## Abstract

**Background:**

Tinnitus is a highly prevalent symptom affecting 10–15% of the adult population. It often affects patient quality of life and frequently causes distress. When subjective tinnitus can be elicited by the somatosensory system of the cervical spine or temporomandibular area it is termed somatic tinnitus. The first aim of the current study is to investigate the effect of the best evidence conservative temporomandibular disorder (TMD) treatment on tinnitus in patients with co-existence of tinnitus and TMD or oral parafunctions compared to no treatment. The second aim is to identify a subgroup of patients with tinnitus that benefits from the conservative temporomandibular joint treatment.

**Methods and design:**

This study is a randomised controlled trial with a delayed treatment design. Patients with a TMD (TMD pain screener ≥ 3 points) or oral parafunctions (such as clenching and bruxism), who are suffering from moderate to severe subjective tinnitus (Tinnitus Functional Index (TFI) between 25 and 90 points), will be recruited from the tertiary tinnitus clinic of the University Hospital of Antwerp, Edegem, Belgium.

Patients will be excluded in case of clear otological or neurological causes of the tinnitus, progressive middle ear pathology, intracranial pathology, traumatic cervical spine or temporomandibular injury in the past 6 months, severe depression as diagnosed by a psychologist, tumours, previous surgery in the orofacial area, substance abuse that may affect the outcome measures, any contra-indication for physical therapy treatment directed to the orofacial area or when they received TMD treatment in the past 2 months.

After screening for eligibility, baseline data among which scores on the TFI, tinnitus questionnaire (TQ), mean tinnitus loudness as measured with visual analogue scale (VAS), TMD pain screener, and a set of temporomandibular joint tests will be collected.

Patients will be randomised in an early-start group and in a delayed-start group of therapy by 9 weeks. Patients will receive conservative TMD treatment with a maximum of 18 sessions within 9 weeks. At baseline (week 0), at the start of therapy (weeks 0 or 9), 9 weeks after therapy (weeks 9 or 18), and at follow-up (weeks 18 or 27) data from the TFI, TQ, VAS mean tinnitus loudness and the TMD pain screener will be collected.

**Discussion:**

Herein, we aim to improve the quality of care for patients with tinnitus attributed to TMD or oral parafunctions. By evaluating the effect of state-of-the-art TMD treatment on tinnitus complaints, we can investigate the usefulness of TMD treatment in patients with somatic tinnitus.

**Trial registration:**

3 July 2017, version 1 of the protocol, ClinicalTrials.gov NCT03209297.

**Electronic supplementary material:**

The online version of this article (10.1186/s13063-018-2903-1) contains supplementary material, which is available to authorized users.

## Background

Tinnitus is a common symptom that occurs in 10–15% of the adult population, often affecting patient quality of life and frequently causing distress [1, 2]. In the absence of any acoustic stimulus (internal nor external) it is termed subjective tinnitus, which is the most common form [1]. Besides hearing loss or noise trauma, tinnitus can also be attributed to the somatic system of the cervical spine or temporomandibular area [1, 3, 4]. This type of tinnitus is termed somatic or somatosensory tinnitus and has been described in 36–43% of a population with subjective tinnitus [5, 6]. The frequent co-existence of tinnitus and temporomandibular disorders (TMD) has been shown in several studies [7, 8]. Manfredini et al. [9] investigated patients with TMD and found a tinnitus prevalence of 30.4%. Furthermore, Lam et al. [10] found that 64% of patients with tinnitus suffered from TMD and Buergers et al. [11] demonstrated that tinnitus is eight times more prevalent in patients with TMD, compared to patients without, and that the tinnitus perception can often be altered by forceful clenching of the teeth.

A physiological explanation for the frequent co-existence is delivered by several animal studies, which have found connections between the somatosensory system of the cervical spine and temporomandibular area on the one hand and the cochlear nuclei (CN) on the other hand [12, 13]. Cervical and temporomandibular somatosensory information is conveyed to the brain by afferent fibres, the cell bodies of which are located in the dorsal root ganglia or the trigeminal ganglion. Some of these afferent fibres also project to the central auditory system and more specifically to the dorsal CN [14]. This makes the somatosensory system able to influence the auditory system by altering the spontaneous rates (i.e. not driven by auditory stimuli) or the synchrony of firing among neurons in the CN, inferior colliculus or auditory cortex. In this way, the somatosensory system is able to alter the intensity and the character of the tinnitus by, for instance, forceful muscle contractions of the neck or jaw musculature or by increased muscle tension in the tensor tympani muscle [15]. These findings have led to the assumption that appropriate treatment of TMD can also alleviate the perceived tinnitus.

An evidence-based conservative management of TMD should focus on the multifactorial aetiology of TMD. Since biological, psychological and social factors may play a role in the aetiology and continuation of TMD, the treatment of TMD should also be based on a multidisciplinary approach [16]. Furthermore, the first line management in all patients with TMD should be to encourage self-management through patient education [17]. Patients should be educated regarding the possible causes of TMD and it is important that they understand their own central role in the management of TMD [17–20]. Depending on the diagnosis and aetiology, the therapy should be individually tailored. Both dentists and physical therapists may play a role in the conservative, individual and multimodal management of such patients [21, 22].

The primary aim of this study is to investigate the effect of a state-of-the-art, conservative temporomandibular joint (TMJ) treatment, as described above, on tinnitus complaints, compared to no treatment. Secondary, this study aims to identify mediating factors, i.e. factors that contribute to the therapeutic effect. To help clinicians in their clinical process, we will identify prognostic indicators, i.e. factors that predict a positive or negative outcome of TMD treatment.

## Methods

### Patients

Patients will be recruited from the Antwerp University Hospital, Edegem, Belgium, by otolaryngologists at their tertiary tinnitus clinic. During this consult, patients will be thoroughly tested to exclude any objective causes of the tinnitus.

All patients will be assessed by means of medical history, ear-nose-throat (ENT) examination with micro-otoscopy, brain magnetic resonance imaging to exclude vascular compression or tumoral processes like acoustic neurinoma, audiometry (pure tone audiometry, tinnitus pitch and loudness matching and speech in noise test), tinnitus assessment comprising tinnitus loudness using a Visual Analogue Scale (VAS), tinnitus annoyance using the Tinnitus Questionnaire (TQ) and tinnitus severity using the Tinnitus Functional Index (TFI). Complementary, all the patients will undergo an electroencephalogram in which the auditory event-related potentials will be measured to investigate the alteration in processing of sound before and after TMJ treatment in an objective way [23].

Patients will be included when suffering from chronic somatic tinnitus, attributed to TMD or oral parafunctions, and which has been stable for at least 3 months. This diagnosis will be made by the ENT surgeon based on the abovementioned clinical process and using the diagnostic criteria for tinnitus attributed to TMD [24]. According to these criteria, tinnitus can be attributed to TMD or oral parafunctions when one of the following criteria are present, namely tinnitus association with manipulation of the teeth or jaw, temporal coincidence of onset or increase of both TMD pain and tinnitus, increase of tinnitus during inadequate postures when resting, walking, working or sleeping, or intense bruxism and/or clenching periods during the day or night (Table 1).

**Table 1.** Inclusion and exclusion criteria

Inclusion	Exclusion
Subjective tinnitus > 3 months	Clear otological or neurological causes of the tinnitus
AND one of the following present:	Severe depression
• Tinnitus association with manipulation of the teeth or jaw	Progressive middle ear pathology
• Temporal coincidence of onset or increase of both TMD pain and tinnitus	Intracranial pathology
• Increase of tinnitus during inadequate postures during rest, walking, working or sleeping	Traumatic cervical spine or temporomandibular injury in the past 6 months
• Intense bruxism and/or clenching periods during the day or night	Tumours
	Previous surgery in the orofacial area
	Substance abuse that may affect the outcome measures
	TMD treatment is contra-indicated
	Already received TMD treatment in past 2 months

*TMD* temporomandibular disorders

Additional evaluations in the context of this study involve temporomandibular assessment. During anamnesis, patients are questioned about the presence of bruxism and clenching. Furthermore, patients are screened on the presence of a painful TMD by the TMD pain screener [25]. In case a patient scores positive on at least three out of six questions of the TMD pain screener, the patient is suspected to suffer from a painful TMD (sensitivity 0.99 and specificity 0.95–0.98, respectively). The patient is then referred to the dentist, who will perform a clinical investigation according to the internationally recognised classification system for TMD [26].

Patients will be excluded in case of clear otological or neurological causes of the tinnitus such as Menière’s disease, severe depression (diagnosed by a psychiatrist), progressive middle ear pathology, intracranial pathology, traumatic cervical spine or temporomandibular injury in the past 6 months, tumours, previous surgery in the orofacial area, substance abuse that may affect the outcome measures or in case physical therapy treatment directed to the orofacial area is contra-indicated. Given the treatment that is studied, patients will also be excluded if they received TMD treatment in the past 2 months (Table 1).

### Study design

**Fig. 1:**
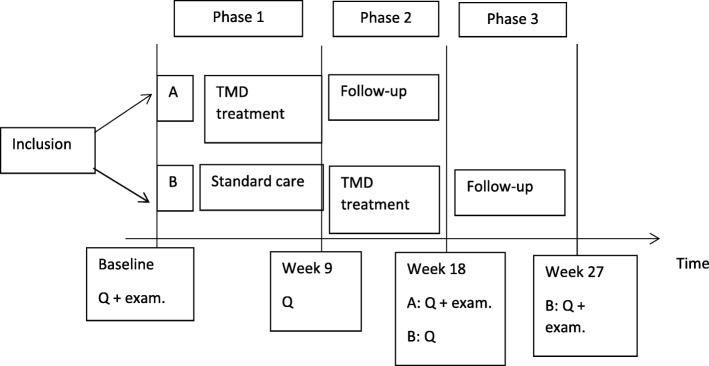
Delayed-start design (A: early-start group; B: delayed-start group; Q: Questionnaires; Exam.: full examination)

This is a randomised controlled trial with a delayed treatment design (Fig. 1). At baseline, patients are randomly assigned by an independent researcher to the early-start group or to the delayed-start group. In part 1, the early-start group receives the TMD treatment for 9 weeks. The delayed-start group receives the standard information and advice about tinnitus, but no treatment in the first 9 weeks. In part 2, the patients in the delayed-start group receive TMJ treatment for the next 9 weeks. The early-start group now enters a follow-up period. In part 3, all patients enter a follow-up period. Follow-up data are collected after 18 and 27 weeks, in line with previous studies about somatic tinnitus of our research group [6, 27].

The results of the trial will be reported according to the CONSORT guidelines.

### Randomisation procedure

Patients will be randomised into the early-start group or into the delayed-start group in a 1:1 ratio insuring an even distribution based on sex and age. The randomised list will be generated by an independent researcher using QMinim Online Minimisation® [28]. The assessor performing the clinical tests is blinded to the allocation of the patients in the direct-start or delayed-start group. This is possible because all patients are investigated 18 weeks after the start of their therapy (Fig. 1). The extra measurements in the delayed-start group, in week 18 of the study, are questionnaires that are completed via an online tool. Patients cannot be blinded in this protocol.

### Outcome measures

#### Primary outcome measure

The primary outcome measure is change in tinnitus-related distress, measured using the Dutch version of the TQ, validated in 2007 [29, 30]. The TQ consists of 52 questions, of which 40 are used for calculating the total score and 2 are counted double (items 5 and 20). The questions are answered on a 3-point scale, ranging from ‘true’ (scoring 0) to ‘partly true’ (scoring 1) to ‘not true’ (scoring 2). The total score on the TQ ranges from 0 to 84. Higher scores correspond with higher tinnitus-related distress. The TQ shows high internal consistency (Cronbach’s alpha: 0.95) and a good correlation with the Tinnitus Handicap Inventory and Tinnitus Impairment Questionnaire (0.83–0.90) [31]. A decrease of 8.72 points is considered as clinically relevant (standardised response mean 1.04) [31].

#### Secondary outcome measures

Various secondary outcome measures will be measured and used to describe the population and identify possible mediating factors, i.e. factors that contribute to the therapeutic effect. An overview is presented in Table 2.

**Table 2:** Overview of primary and secondary outcome measures

	Baseline	Follow-up	Measuring tool	Completed by
Primary outcome measure				
Tinnitus-related distress	X	X	Tinnitus Questionnaire	Patient
Secondary outcome measure				
TMJ pain	X	X	TMD pain screener	Patient
Myalgia	X	X	DC/TMD	Researcher
Arthralgia	X	X	DC/ TMD	Researcher
Pressure sensitivity	X	X	Pressure algometer (Somedic), kPa	Researcher
Mouth opening	X	X	Ruler, cm	Researcher
Hearing loss	X		Pure Tone audiometry Speech in noise test	Audiologist
Psychoacoustic tinnitus analysis	X	X	Type of tinnitus, tinnitus pitch and tinnitus loudness	Audiologist
Tinnitus severity	X		Tinnitus Functional Index	Patient
Subjective tinnitus loudness	X	X	Visual Analogue Scale (0–100 mm)	Patient
State of anxiety and depression	X		Hospital Anxiety and Depression Scale	Patient
Hyperacusis	X		Hyperacusis Questionnaire	Patient
Auditory event-related potential	X	X	Auditory event-related potential	Researcher
General tinnitus characteristics such as duration of the complaints, localisation (unilateral, bilateral, central), somatic modulation, temporal relation with TMD, bruxism periods	X		Medical history	ENT

*DC/TMD* Diagnostic criteria for temporomandibular disorders, *ENT* ear-nose-throat, *TMD* temporomandibular disorders, *TMJ* temporomandibular joint

##### Temporomandibular outcome measures

The TMD pain screener is a 6-item questionnaire regarding pain complaints from the orofacial region, and their dependency on functions, like opening wide or chewing. Internal consistency of the questionnaire is excellent, with coefficient α value of 0.93, reliability is acceptable (intraclass correlation coefficient 0.79), and it has excellent sensitivity and specificity (0.99 and 0.95–0.98, respectively) [25].

Apart from the questionnaire, a set of clinical TMD tests are performed to investigate the presence of a painful TMD. The examination includes the following tests:

- The assessment of pain on active movements of the mandible, palpation of the jaw muscles and temporomandibular joint, and measuring of the mouth opening. This will be performed according to the standardised protocol of the Diagnostic Criteria/TMD [26]. Based on this examination, the presence of the specific TMD-pain diagnosis will be assessed as myalgia (sensitivity 0.90, specificity 0.99) and/or arthralgia (sensitivity 0.89, specificity 0.98).
- Pressure pain thresholds (PPTs) will be measured using a hand-held algometer (Somedic AB, Farsta, Sweden). The PPTs will be measured on the temple, the masseter and the sternocleidomastoid muscles, and on the TMJ. The tibialis anterior muscle will be used as a reference point. The PPTs are expressed in kPa. The average of three measurements will be used for further calculations. For the measurement of PPTs, the pressure is progressively increased. Subjects have to report when the feeling of pressure changes into a feeling of pressure and pain by pressing a patient-controlled switch. Measuring pressure pain thresholds on masticatory muscles and the temporomandibular joint has been shown to be accurate and reliable [32].

##### Audiological outcome measures

The TFI [33] assesses tinnitus severity focusing on eight different domains, namely the unpleasantness of the tinnitus, reduced sense of control, cognitive interference, sleep disturbance, auditory difficulties attributed to the tinnitus, interference with relaxation, reduction in quality of life and emotional distress. The test-retest reliability of the TFI is good (r = 0.78). The convergent validity with the Tinnitus Handicap Inventory (r = 0.86) and VAS (r = 0.75) is good, as well as the discriminant validity with the Beck Depression Inventory-Primary Care (r = 0.56). A reduction of 13 points is considered to be clinically relevant. This questionnaire will be used to obtain a detailed view on the patients’ subjective tinnitus complaints.

The subjective loudness of the tinnitus is rated using a VAS. The patient is asked to indicate the average loudness of their tinnitus on a 10 cm horizontal line. On this line, the left end indicates ‘no tinnitus’ and the right end indicates ‘as loud as you can imagine’.

The Hospital Anxiety and Depression Scale (HADS) is a self-assessment scale developed to identify the possibility and probability of the presence of anxiety and depression among patients in non-psychiatric hospital clinics [34]. It consists of two subscales, an Anxiety subscale (HADS-A) and a Depression subscale (HADS-D), both containing seven intermingled items. All symptoms of anxiety or depression relating also to physical disorder, such as dizziness, headaches, insomnia, anergia and fatigue, are excluded to prevent intrusion from somatic disorders on the scores. Since symptoms relating to serious mental disorders were less common in patients attending a non-psychiatric hospital clinic, these symptoms are also excluded [34, 35]. The HADS has been found to be a reliable instrument for detecting states of depression and anxiety in the setting of a hospital medical outpatient clinic [34].

Hyperacusis is quantified and characterised using the Dutch version of the Hyperacusis Questionnaire (HQ) [36]. The HQ consists of 14 questions answered on a 4-point scale, ranging from ‘No’ (scoring 0 points), ‘Yes, a little’ (scoring 1 point), ‘Yes, quite a lot’ (scoring 2 points) to ‘Yes, a lot’ (scoring 3 points). Scores on the HQ consequently range from 0 to 42 and the cut-off value for hyperacusis is 28 points [37]. The HQ is used to investigate the presence of hyperacusis.

In addition to the questionnaires, a set of audiological parameters will be performed, comprising pure tone audiometry, tinnitus analysis and speech in noise test.

- Pure tone audiometry, the key hearing test used to identify hearing threshold levels, will be measured according to the current clinical standards (ISO 8253-1, 1989), using a two-channel Interacoustics AC-40 audiometer in a soundproof booth. Air conduction thresholds will be measured at 125 Hz, 250 Hz, 500 Hz, 1 kHz, 2 kHz, 3 kHz, 4 kHz, 6 kHz and 8 kHz. This test is used to identify the presence and level of hearing loss. When the level of hearing loss corresponds to the tinnitus pitch, a causal relation can be expected [38].
- The tinnitus analysis starts with identifying the type of tinnitus by asking whether one perceives a pulsatile or non-pulsatile tinnitus, whether the tinnitus is perceived constantly or not and whether the tinnitus sound is a pure tone, a noise or a mixture of different sounds (polyphonic); the tinnitus pitch is then assessed. The pitch is the psychoacoustic equivalent of the physical parameter frequency and is obtained by use of a pitch-matching technique that is the quantitative and qualitative description of the spectral characteristics of the tinnitus. For this technique, a two-alternative forced choice procedure is employed, using the contralateral ear as the reference ear. In cases where tinnitus is perceived bilaterally, the choice of ear is arbitrary. Using this technique, an attempt is made to identify the centre pitch of the tinnitus. When multiple tinnitus sounds are perceived, it is suggested to concentrate on the most troublesome tinnitus sound. Each time a pair of pure tones, or noises in case of noise-like tinnitus, differing by one or more octaves, are presented to the subject, who has to indicate which of the tones resembles the tinnitus the most. This procedure is repeated and finer adjustments are made to obtain the most exact match of tinnitus pitch possible. Afterwards, a tinnitus loudness matching is performed. Loudness is the perceptual correlate of the sound intensity. The tone (or noise) defined as the pitch match is presented to the ipsilateral ear when appropriate and a loudness match is made by use of an alternating procedure. Because of the compressed dynamic range frequently present at the tinnitus frequency, final loudness measurements are made with 1 dB steps. This measures the absolute level of tinnitus loudness expressed in dB hearing level. In addition, a calculation is made to provide a measurement of relative loudness expressed in dB sensation level, that is, the level of the loudness match minus the auditory threshold at tinnitus frequency. The tinnitus analysis provides an objective image of the perceived sound [38].
- Speech in noise tests are performed to investigate whether or not patients with somatic tinnitus attributed to TMD, in whom no hearing loss is assumed, perform worse on speech in noise tests. The Leuven Intelligibility Sentence Test [39], a Dutch sentence test, will be applied; it consists of 35 lists of 10 sentences that are a reflection of daily communication and are of equivalent difficulty. An adaptive procedure is used with the noise at a fixed level of 65 dB sound pressure level. The procedure starts at a signal-to-noise ratio of 0 dB, meaning that speech and noise are presented equally loud (65 dB sound pressure level). Subsequently, the intensity level within a list of sentences is varied in steps of 2 dB adaptively in a one-down (when the keywords in the sentence are correctly repeated), one-up (when the keywords in the sentence are incorrectly repeated) procedure to determine the 50% correct identification point, which is called the speech reception threshold, expressed in dB signal-to-noise ratio. Before starting the actual procedure, one list will be performed as a training list for both left and right ear.

Additionally, all patients will undergo an electroencephalogram in which the auditory event-related potentials will be measured to investigate the alteration in processing of sound before and after TMJ treatment in an objective way [23]. This provides an objective outcome measure besides the subjective outcome measures.

Finally, the information from medical history taking by an ENT doctor, who is part of the standard tinnitus assessment, is used to gain insight into the duration of the complaints. Questions referring to the diagnostic criteria of somatic tinnitus are also included in the anamnesis, including duration of the complaints, localisation of the tinnitus (unilateral, bilateral or central), tinnitus association with some manipulation of the teeth or jaw, temporal coincidence of appearance or increase of both pain and tinnitus, increase of tinnitus during inadequate postures during rest, walking, working or sleeping, bruxism and/or clenching periods during day or night. In Table 2, an overview of the primary and secondary outcome measures is presented.

### Intervention

TMD treatment is multifactorial, and is provided by dentists and physical therapists. It consists of patient education on normal jaw function, avoidance of overuse in oral ‘bad habits’ such as nail biting and tooth clenching. In case of grinding, night time use of stabilisation splints can be applied. Bothersome malocclusions will be addressed, e.g. by providing stabilisation splints. Patients are encouraged to relax their masticatory muscles, and relaxation of the muscles will be trained. Painful muscles will be stretched, wherein stretching techniques will be instructed to the patients so they can continue these exercises at home. During every physical therapy session, these exercises will be boosted to enhance patient compliance [40]. In total, the treatment period can last up to 9 weeks. This is longer than the traditional 6 weeks, yet it is based on our previous trial concerning cervicogenic somatic tinnitus, where an increase of complaints was observed after 6 weeks of therapy [6]. We therefore provide 3 additional weeks where the patient can be followed-up by the treating healthcare professional. Depending on the needs, up to 18 physical therapy sessions can be scheduled over a period of 9 weeks; two sessions per week for the first 3 weeks, one session per week for the next 6 weeks, and optional additional sessions to monitor patient compliance.

Trained clinicians will provide treatment. All participating clinicians are trained in the treatment protocol by the researchers. Patients with tinnitus attributed to a TMD will be referred to the trained clinicians for TMD treatment (guided referral).

### Sample size and power

The primary outcome measure, the TQ, will be used to investigate differences between the direct-start group and delayed-start group in week 9 of the study. We plan to include patients in both groups in a 1:1 ratio. Based on literature data [31], we expect a minimal significant decrease in TQ score of 8.72 points (SD 13.72). To be able to reject the null hypothesis that the population means of the direct-start group and delayed-start group in week 9 are equal with probability (power) of 0.8, we will need to study 37 patients in each group. The Type I error probability associated with this test of this null hypothesis is 0.05.

This sample size will be refined after a pilot study containing 2 × 5 patients. Considering the possibility of dropouts and the later analysis of prognostic indicators and mediation analysis, we aim to recruit 2 × 100 patients.

The primary analysis population is the intention-to-treat population. This population includes all randomised patients who provided baseline data, regardless of whether or not they adhere to the complete protocol.

### Statistics

Our null hypothesis is that the change in TQ at week 9 is equal in both groups (early-start and delayed-start):

H_0_: Change in TQ-baseline to TQ-9 weeks (early start) = Change in TQ-baseline to TQ-9 weeks (delayed start).

The primary outcome is a change in the scores on the TQ after 9 weeks of treatment. The mean change in TQ score between baseline and 9-week (post treatment) scores will be calculated. This mean change in TQ of the early start group will be compared to the mean change in TQ of the delayed-start group that has not yet received treatment at this point. A difference between both groups will be defined as statistically significant when *p* < 0.05.

A repeated measures ANOVA and post-hoc tests will be used to compare the mean changes of the early-start group and delayed-start group at 9 weeks, and secondary at baseline, 18, and 27 weeks follow-up. The same cut-off point of *p* < 0.05 will be used here.

For the mediation analysis, the protocol described by Baron and Kenny [41] will be used to calculate the role of potential contributing factors in the obtained output. This implies the execution of three regression analyses. The first will assess the relation between the applied therapy (independent variable) and the outcome (dependent variable). The second will assess the relation between the applied therapy (independent) and a mediating factor to confirm that the applied treatment significantly affects the mediator. The third will be used to confirm that the mediator significantly predicts the treatment outcome (dependent variable), while controlling for the applied treatment (independent variable). Again, a cut-off point of *p* < 0.05 will be used.

Bootstrapping will be performed to avoid overestimation of the mediation effect. As potential mediating factors, all secondary outcome measures will be used.

## Discussion

The aim of this study is to investigate the effect of a conservative TMJ treatment on several tinnitus and TMJ-related parameters. Currently, the studies that investigated the effect of TMJ therapy on somatic tinnitus have a high risk of bias, mainly due to a lack of statistical analyses between groups and before-after treatment, incomplete presentation of the data and selective reporting [42]. Additionally, risk of bias is present due to a lack of information about the blinding process of the subjects, therapists and investigators. Blinding of the subjects and therapists is always a hurdle in studies investigating therapy treatment and is hard to overcome. Therefore, blinding of the assessor, who performs the follow-up measurements and data processing, is even more crucial. In this study, a high-quality methodological design will be used by performing a prospective comparative delayed design with a blinded evaluator at baseline, end of therapy, and 9 and 18 weeks after therapy.

Furthermore, earlier studies that investigated the effect of conservative TMJ treatment on somatic tinnitus did not always match the evidence-based practice for TMJ treatment. In patients with tinnitus as well as TMD or oral parafunctions, it is thought that tinnitus improvement can be achieved by improving the TMJ complaints. Therefore, it is necessary to use the best available TMJ treatment option in order to gain maximal improvement in tinnitus complaints. For example, Bösel et al. [43] applied self-therapy next to splint therapy, although Tuncer et al. [44] found that physiotherapy performed by a therapist in combination with home physical therapy was more effective in terms of TMD pain and pain-free maximal mouth opening than home physical therapy alone.

Additionally, the multifactorial aetiology of TMD should be considered. Studies have proven that TMD patients show increased somatisation, stress, anxiety and depression compared to healthy individuals [45, 46]. Therefore, there is a need for multimodal therapies, incorporating behavioural and educational approaches, which seem to offer more benefit than a single-treatment program in patients with high psychological distress [47].

In this respect, we decided to use a multimodal treatment in the current study, which is provided by dentists and physical therapists. All physical therapists are trained in the treatment protocol by the researchers (guided referral) and will adjust the treatment modalities to the needs of the individual patient.

Currently, the clinical evaluation of somatic tinnitus is based on a generally accepted set of anamnestic criteria [24]. According to these criteria, somatic tinnitus is suspected when the tinnitus is associated with an evident history of head or neck trauma, some manipulation of the teeth, jaw or cervical spine, recurrent pain episodes in head, neck or shoulder girdle, temporal coincidence of onset or increase of both pain and tinnitus, increase of tinnitus during inadequate postures when resting, walking, working or sleeping, or intense bruxism periods during the day or night. These criteria imply a temporal and mechanical association between TMJ or cervical spine dysfunction, and tinnitus complaints. If one of the abovementioned criteria is present, somatic tinnitus is suspected and further evaluation is warranted. Other studies have described the presence of modulation of tinnitus during forceful contractions of the neck and jaw musculature [5, 48]. However, these tests also elicit a sound perception in 65% of an asymptomatic control group without tinnitus complaints, which makes modulation tests minimally useful for diagnosing somatic tinnitus. Therefore, we apply a combination of thorough ENT, audiological and temporomandibular assessments combined with the abovementioned diagnostic criteria to diagnose somatic tinnitus. While all objective causes of tinnitus are excluded, we aim to include patients with a co-existence of severe subjective tinnitus and temporomandibular complaints in order to include patients who can potentially benefit from the treatment.

To obtain a detailed view on the patients’ subjective tinnitus complaints, the TFI will be used [33]. Since the responsiveness of this questionnaire is limited due to important floor effects on more than half of the questions, this questionnaire will not be used to evaluate treatment effect [49].

To improve the quality of patient care, it is important that clinicians are able to identify who has a high likelihood for a positive treatment effect and who is at risk of poor recovery. Therefore, we aim to identify prognostic indicators to provide arguments why someone might benefit from TMJ treatment or not. This can reduce the current trial and error strategy and thus avoid patient frustration and unnecessary reimbursement of unsuccessful therapies.

To date, no prognostic indicators are available for the effect of TMD treatment on tinnitus. However, an earlier study in cervicogenic somatic tinnitus showed that patients with low-pitched tinnitus, that co-varies with the neck complaints and increases during inadequate postures, has the best prognosis after cervical spine treatment [50]. Rollman et al. [51] studied a variety of prognostic indicators for TMD pain in general and found that a longer duration of the TMD-pain complaint, a higher number of attended care practitioners and higher degree of hindrance on function, negatively predicted 6-month improvement. It is unclear to what extent these prognostic indicators can be transferred to our population of patients with tinnitus as a main complaint; consequently, we aim to identify prognostic indicators for the effect of TMJ treatment on tinnitus.

To understand the working mechanism behind TMJ treatment for somatic tinnitus it is necessary to identify variables that have an effect on the main outcome variable (TQ). Knowing that a treatment is beneficial for patients is important, but knowing how it works adds to the understanding and acceptance of the findings among clinicians. Therefore, we also aim to identify possible mediating factors, i.e. factors that contribute to the therapeutic effect. The underlying idea is that TMJ contributes to somatic tinnitus. Consequently, variables that measure TMJ parameters are used as potential mediators; these are TMJ pain via the TMD pain screener, pain sensitivity as measured via PPT and mouth opening.

Since patients are recruited in a tertiary centre with long waiting lists, it would be unethical to include patients in a placebo group receiving no treatment at all. Therefore, this study is designed as a randomised controlled trial with a delayed treatment design. In this type of design, one group of patients will receive 9 weeks of treatment immediately, while the delayed-start group will receive the standard information and advice about tinnitus. After 9 weeks, the delayed-start group will also receive the TMD therapy.

To date, the inclusion of the pilot study is completed. The follow-up results are expected by the end of December 2017. Afterwards, a sample size recalculation will be performed.

This study is the first to investigate the effect of a state of the art conservative TMJ treatment protocol on patients with somatic tinnitus using a prospective comparative delayed design and blinded evaluator for baseline, end of therapy, and 18 and 27 weeks after therapy (Additional file 1).

### Trial status

The study is in the recruitment phase.

## Additional file

Additional file 1: SPIRIT 2013 Checklist: Recommended items to address in a clinical trial protocol and related documents. (PDF 1226 kb)

## Abbreviations

CN: cochlear nuclei
ENT: ear-nose-throat
HADS: Hospital Anxiety and Depression Scale
HQ: Hyperacusis Questionnaire
PPT: pain pressure threshold
TFI: Tinnitus Functional Index
TMD: temporomandibular disorders
TMJ: temporomandibular joint
TQ: Tinnitus Questionnaire
VAS: Visual Analogue Scale
